# Temozolomide – Just a Radiosensitizer?

**DOI:** 10.3389/fonc.2022.912821

**Published:** 2022-06-16

**Authors:** Bernd Kaina, Lea Beltzig, Herwig Strik

**Affiliations:** ^1^ Institute of Toxicology, University Medical Center, Mainz, Germany; ^2^ Department of Neurology, Sozialstiftung, Bamberg, Germany

**Keywords:** glioblastoma, temozolomide, radiotherapy, MGMT, DNA repair, apoptosis, treatment protocols

## Abstract

Radiation concomitant with the DNA methylating drug temozolomide (TMZ) is the gold standard in the treatment of glioblastoma. In this adjuvant setting, TMZ is regarded to be a radiation sensitizer. However, similar to ionising radiation, TMZ induces DNA double-strand breaks and is itself a potent trigger of apoptosis, cellular senescence and autophagy, suggesting that radiation and TMZ act independently. Although cell culture experiments yielded heterogeneous results, some data indicate that the cytotoxic effect of radiation was only enhanced when TMZ was given before radiation treatment. Based on the molecular mechanism of action of TMZ, the importance of specific TMZ and radiation-induced DNA lesions, their repair as well as their interactions, possible scenarios for an additive or synergistic effect of TMZ and radiation are discussed, and suggestions for an optimal timing of radio-chemical treatments are proposed.

## Introduction

Temozolomide (TMZ), an orally deliverable alkylating chemotherapeutic agent, is essential part of the standard treatment of glioblastoma (GBM). It is applied after tumor resection in a dose of 75 mg/m^2^ once per day, concomitantly with fractionated ionizing radiation (IR), with 2 Gy per fraction, cumulating up to 60 Gy (1). After radio-chemotherapy, adjuvant treatment consists of TMZ at higher dose (150-200 mg/m^2^, six cycles day 1-5/28) (1). At relapse, several modified protocols are applied that deviate from this classic “Stupp” regimen (2). Dose-dense schedules consist of TMZ at lower doses, i.e. 150 mg/m^2^ day 1-7/14, 100 mg/m^2^ day 1-21/28, 100 mg/m^2^ day 1-5/7 or 50 mg/m^2^ administered continuously (3). Even at these low dose levels, TMZ is effective, as indicated by clinical studies (4, 5). Moreover, at least in O^6^-methylguanine-DNA methyltransferase (MGMT)-deficient tumors, repeated TMZ treatments very likely lead to an accumulation of critical DNA damages that trigger cell death, which is supported by our studies on glioblastoma cells *in vitro* (6). The concurrent administration of TMZ with IR results in a prolongation of overall survival (OS) and progression free survival (PFS) by a few months (1).

An alternative therapy regimen is based on lomustine (CCNU), a chloroethylating agent that induces O^6^-chloroethylguanine and subsequently interstrand-crosslinks in the DNA (7). It is administered at day 1, followed by TMZ day 2-6 of the 6-week cycles. In this setting, the PFS is identical to the Stupp protocol, but the OS is increased, albeit at the expense of additional side effects (8). In this setting, IR is not included even though it is conceivable that IR together with lomustine ameliorates glioblastoma cell death, given the cell death pathway triggered by chloroethylating agents (7). However, it is expected that the side effects in the irradiation field are more severe since the toxicity of lomustine and IR is likely not restricted to the proliferating tumor cell pool, but also to the non-proliferating healthy brain tissue. In contrast, the toxic effect of TMZ is strictly limited on replicating cells (9) and, therefore, specifically affects the proliferating tumor cell population. Nearly nothing is known about the complex interaction of TMZ, IR and lomustine-induced DNA damage and signaling.

## Tmz and Radiation

**Figure 1:**
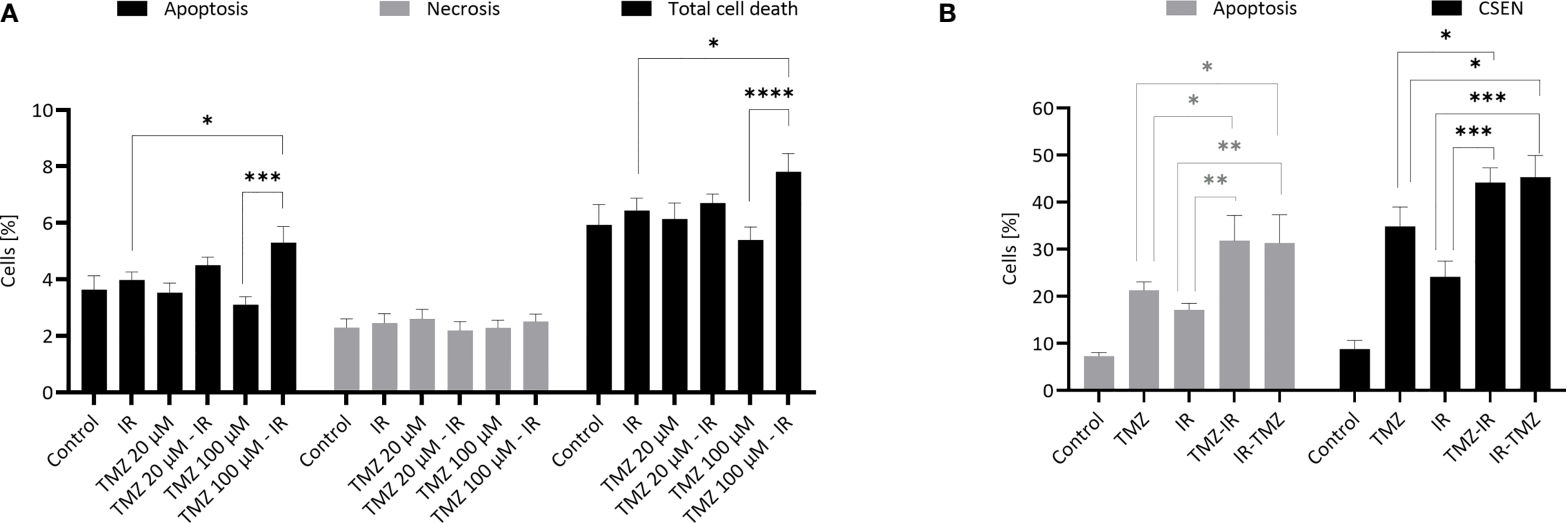
TMZ and IR- induced apoptosis and senescence. **(A)** Effect of TMZ pretreatment on radiation-induced apoptosis. Human LN229 glioblastoma cells in the exponential growth phase were treated with TMZ (20 or 100 µM) and 3 h later irradiated with γ-rays (6 Gy). Apoptosis (annexin V+, PI-), late apoptosis/necrosis (annexin V+, PI+) and total cell death were measured 5 h later by flow cytometry. Data are the mean of 4 independent experiments +/- SEM. *p < 0.05; ***< 0.001. **(B)** Effect of IR pre- and post-treatment on TMZ-induced apoptosis and cellular senescence. Human LN229 glioblastoma cells in the exponential growth phase were treated with TMZ (20 µM) and 3 h later irradiated with γ-rays (6 Gy) (TMZ-IR). In a parallel setting, they were irradiated (6 Gy) and 6 h later treated with TMZ (20 µM) (IR-TMZ). Apoptosis (early apoptosis: annexin V+/PI-; late apoptosis: annexin V+/PI+) and senescence (C12FDG+) were measured 7 d later by flow cytometry. Data are the mean of 3-4 independent experiments +/- SEM. *p < 0.05; **< 0.01; ***< 0.001 ; ****<0.0001. Experiments were essentially performed as previously described (16).

In chemoradiation, TMZ is believed to be a radiosensitizer (10). This view is based on a series of experimental studies which, taken together, led to the conclusion that pretreatment with TMZ or simultaneous treatment with TMZ and IR increases cytotoxicity above the additive effect. Actually, these studies are quite heterogeneous, ranging from additive to near-synergistic effects (11–15). Thus, treatment with a low dose of TMZ 2 h before irradiation exacerbated the cytotoxic effects that were described as supra-additive, which was not the case when TMZ was administered after IR (11). We repeated this experiment and were able to confirm that TMZ pretreatment enhances the level of IR-induced apoptosis. However, this was only the case if a high dose of TMZ was used (100 µM instead of 20 µM). It is important to note that in this setting IR (6 Gy) and TMZ alone did not enhance apoptosis (which was measured 5 h after IR treatment) above the control level (**Figure 1A**).

Apoptosis induced by TMZ in glioblastoma cells is a late response, starting 3 d after TMZ treatment and reaching a maximum 5 d later, while senescence reaches a plateau after 7 d (17). To see whether IR has an impact on TMZ-induced cell death, we treated glioblastoma cells with a therapeutically relevant low dose of 20 µM TMZ 2 h before or 6 h after IR (6 Gy) and measured apoptosis and cellular senescence (CSEN) 7 days later. Under these conditions, both TMZ and IR alone induced significant apoptosis. Interestingly, TMZ pre- and post-treatment were effective in enhancing the apoptosis level (**Figure 1B**). For the endpoint cellular senescence we also observed, similar to apoptosis, a significant increase (**Figure 1B**). However, the effects were less than additive, indicating that IR provokes rather an inhibiting effect on TMZ-induced genotoxic pathways. Overall, our data support the notion that the timing (pre-treatment) and the TMZ dose are critical as to the amelioration of the cytotoxic effect of IR.

## Tmz - a Radiosensitizer?

Whether TMZ is a real radiosensitizer has not yet been critically questioned. As we know the mechanism of action of TMZ quite precisely (7, 18), it is pertinent to answer this question anew. A genuine radiosensitizer is characterized by provoking an increase in the radiation effect, whereby the radiosensitizer itself is not toxic (19). In tumor therapy, the intended endpoint of the biological radiation effect is death of the tumor cell, with the number of unrepaired DNA double-strand breaks (DSBs) being the critical, decisive factor (20). Theoretically, radiosensitization can occur through the following mechanisms: a) increase in the number of radiation-induced DSBs, b) reduced repair of the induced breaks resulting in an enhanced level of critical breaks, c) amelioration of the DNA damage response that triggers cell death, d) stimulation of pro-apoptotic pathways, e) inhibition of antiapoptotic pathways, f) inhibition of autophagy and cellular senescence, which counteract the cell death response.

Does TMZ meet these requirements? TMZ does not impact the repair of radiation-induced DSBs. However, it is conceivable that the genotoxic properties of TMZ itself contribute to an increase in the amount of toxic DSBs. How this can occur requires a closer look at the mode of action of TMZ. Chemically, the drug is a triazene derivative that does not need metabolic activation. It decomposes spontaneously in the cell yielding 3-methyl-(triazen-1-yl)imidazole-4-carboxamide and, in a second step, 5-aminoimidazole-4-carboxamide and monomethyl hydrazine, which finally methylates all nucleophilic centers in the cell. However, as revealed by studies with DNA repair mutants and isogenic cell lines (21), the main cytotoxic target of TMZ is the nuclear DNA. Similar to other S_N_1 alkylating agents, TMZ alkylates the DNA at 12 nucleophilic sites (22). The major methylation products are N-methylpurines such as N7-methylguanine, N3-methylguanine, and N3-methyladenine (comprising about 80% of total alkylation), whereas base oxygen methylations are less frequent. Thus, O^6^-methylguanine (O^6^MeG) accounts for only 7% of the total DNA alkylation. Although produced in minor amounts, O^6^MeG is the main mutagenic, carcinogenic, genotoxic, and cytotoxic lesion (23). It is also responsible for autophagy and cellular senescence, which are induced by TMZ concomitant to apoptosis and which counteract cell death (17, 24).

TMZ has a short half-life (about 2 h in serum) and therefore exposures can be considered pulse-treatments. O^6^MeG in the DNA arrives a peak level within 3-4 h after TMZ treatment of glioblastoma cells (16). In cells with the DNA repair protein MGMT the damage is repaired within minutes and therefore cannot develop any toxic effects. However, in tumor cells without MGMT, which are classified as promoter-methylated (25), O^6^MeG remains and accumulates following repeated treatments in the tumor cell DNA.

## Molecular Mechanism of Tmz and Ir

The mechanisms of O^6^MeG-triggered cell death responses have been thoroughly investigated (26). O^6^MeG itself is not a cytotoxic DNA damage, but in proliferating cells the lesion results in O^6^MeG/thymine mismatches during DNA replication. These are recognized by the cell’s mismatch repair (MMR) system. The MMR proteins (MSH2, MSH6, MLH1 and PMS2) repair the damage by removing thymine, but the mispairing properties of O^6^MeG results in restoring the mismatch; the repair is on the spot, leading to futile repair cycles. This finally causes gaps in the DNA and, in a subsequent round of replication, inhibition of the DNA synthesis and open replication forks with vulnerable single-stranded DNA, whose cleavage by nucleases inevitably leads to DSBs. This occurs in the post-treatment cell cycle (i.e. two DNA replication cycles after induction of O^6^MeG are required), which is compatible with the time-course of apoptosis and DSB formation (27). Results obtained with synchronized cells confirmed this model (28). It was further shown that DSBs, induced by the processing of O^6^MeG/thymine, trigger complex DNA damage response pathways, which are activated primarily by ATR, and secondary by ATM, and downstream by CHK1 and CHK2, respectively (29), as well as activation of the SIAH1-HIPK2–p53 axis (30). In this scenario, the following factors determine drug resistance: MGMT, MMR, the proliferation level, DNA damage response (DDR) activation, and DSB repair by homologous recombination (HR). Repair of TMZ-induced DSBs by non-homologous end-joining (NHEJ) plays only a marginal role (31, 32). In contrast to TMZ, IR induces DSBs replication independently and the main pathway of DSB repair following IR in G1 is NHEJ (33). Non-repaired IR-induced DSB activate the DDR mainly *via* ATM, while O^6^MeG mediated replication blocks and DSBs activate both ATR and ATM, which trigger downstream cell death pathways (7, 29).

**Figure 2:**
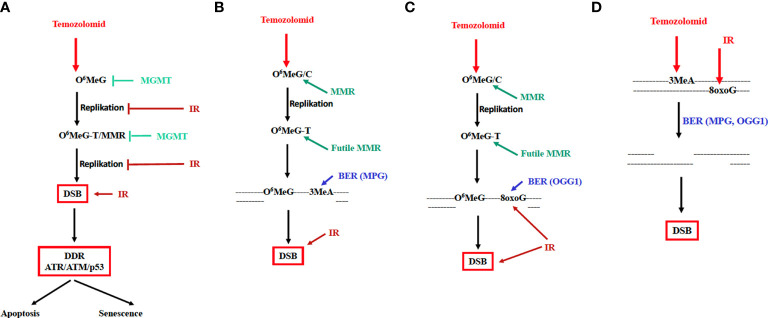
Mechanism of TMZ-induced cytotoxicity and cellular senescence and possible mode of interaction of TMZ and IR. MGMT and IR inhibiting the cytotoxic pathway are indicated. DDR: DNA damage response involving the kinases ATR, ATM and the transcription factor p53. **(A)** Genotoxic pathway triggered by O^6^MeG; futile MMR cycle model. **(B)** MMR and BER of N-methylation lesions cooperate in inducing DSBs. **(C)** MMR and BER of oxidative lesions cooperate in inducing DSBs. **(D)** DSBs are formed in overlapping base excision repair patches of lesions induced by TMZ and radiation. BER, long-patch base excision repair; MPG, N-methylpurine-DNA glycosylase; OGG1, 8-oxo-guanine-DNA glycosylase.

In view of this scenario, it is conceivable that the DSB rate in the tumor cells is increased, and cell death pathways are activated to a greater extent if TMZ is administered together with IR (see **Figure 2A**). This presupposes, however, that a) the tumor cells are MGMT-deficient, b) the tumor cells proliferate (since the conversion of O^6^MeG into DSB is strictly replication-dependent) and c) MMR as well as the DDR are not affected by IR. It should be noted that IR is a potent inducer of genes, which has also been discussed for the *MGMT* gene (34). However, *MGMT* silencing is caused by promoter methylation and IR would therefore have to change the promoter methylation status, which according to available data is not the case. Moreover, we have not been able to demonstrate any *MGMT* induction by IR in glioblastoma cells *in vitro* (35).

The possible influence of IR on mismatch repair, DNA synthesis and tumor cell proliferation is critical. *In vitro*, the MMR genes MSH2 and MSH6 were shown to be downregulated by IR (17). Whether this also occurs *in vivo* is unknown. Radiation inhibits cell cycle progression, arresting cells in the G1/S (36), and dose response curves do not display a no-effect threshold for DNA synthesis inhibition (37). It is therefore conceivable that DNA synthesis is inhibited already by a single therapeutic dose of 2 Gy, and even more so by repeated doses. Under these conditions TMZ will inevitably become ineffective since the conversion of O^6^MeG into DSB cannot take place. According to these considerations, radiation prior to the administration of TMZ would be counterproductive, at least if the radiation dose is sufficiently high to cause DNA synthesis inhibition through G1 blockage (**Figure 2A**). It is conceivable that concurrent daily treatments with 2 Gy plus TMZ may lead to even stronger inhibition of replication and therefore to a significant attenuation of the TMZ-induced cytotoxic response.

## Possible Interactions Between Radiation- and Tmz-Induced Lesions

As already mentioned, experimental evidence suggests that treatment of GBM cells with TMZ + IR can have both synergistic and additive effects. A detailed study showed that dosage and timing are crucial. Using very low doses of TMZ, radiosensitization was only recorded when TMZ was given to the cells before irradiation; TMZ post-treatment had no effect (11). It was concluded that toxic N-alkylations induced by TMZ (such as 3-methyladenine) and O^6^MeG interact, leading to an increase in the DSB rate. A model based on this supposition is shown in **Figure 2B**. This model claims that base excision repair in O^6^MeG/T mismatch repair patches may lead to DSB formation. If one additionally assumes that following irradiation oxidative base damages (such as 8-oxoG or thymine glycol) were generated in the immediate vicinity of O^6^MeG, it is conceivable that MMR of O^6^MeG/T and, concomitantly, BER of oxidative damage (e.g. through the repair enzyme OGG1) in the O^6^MeG harboring strand lead to the formation of DSB (**Figure 2C**).

This model applies to radiation exposure in the TMZ post-treatment cell cycle. It does not explain, however, why pretreatment with TMZ has a radio-sensitizing effect (**Figure 1A**). An explanation for this finding rests on the supposition that base damage through alkylation (N7-MeG, N3-MeG, N3MeA) and oxidation (e.g. 8-oxoguanine) in overlapping BER patches may lead to DSB, similar to what was proposed for extensive alkylation damage in overlapping repair patches (38). Given a patch size of about 25 nucleotides during long-patch BER (39) this likely happens at high DNA alkylation and oxidation levels. Since BER peaks immediately after damage induction, DSB formation in overlapping BER patches is anticipated to occur in the first hours after treatment. This is entirely different from DSBs resulting from O^6^MeG/T processing, which results in a wave of DSB >3 days after TMZ treatment (own unpublished data). Interestingly, apoptosis induced by IR in TMZ pretreated glioblastoma cells can be observed already 5 h after irradiation. It is also important to note that a low dose of TMZ is insufficient; only with a high dose of 100 µM the yield of radiation-induced apoptosis was significantly enhanced (**Figure 1A**). This data confirms what was reported by Bobola et al. (11), and is compatible with the model shown in **Figure 2D**. The observation that TMZ pretreatment leads to radio-sensitization in both MGMT proficient and deficient cells, i.e. independent of O^6^MeG (11), further supports this model. In summary, TMZ appears to be a radiosensitizer when TMZ treatment occurs prior to irradiation.

It is important to note that apart from being a radiosensitizer, TMZ itself is a powerful cytotoxic agent that induces not only apoptosis but also autophagy and cellular senescence (24). Importantly, dose-response studies revealed that O^6^MeG adducts in the DNA, DSBs, apoptosis and senescence increase linearly with dose, without a clear threshold (16, 40). This supports the notion that low doses of TMZ are effective in MGMT lacking tumors. The cytotoxic potency of TMZ leads to the question of whether, conversely, IR can cause an amplification of the TMZ effect, i.e. acting as drug sensitizer. This is conceivable given the cell death cascade evoked by O^6^MeG. In this scenario, blocked replication forks that activate ATR, and DSBs generated through O^6^MeG/T-MMR activating ATM (29) are the decisive downstream events triggering the DDR (**Figure 2A**). If in this window additional DSBs were induced by radiation treatment, it is conceivable that the toxic effect of TMZ is ameliorated. However, own unpublished data showed that no synergistic, but at best only additive effects were recorded under these treatment conditions.

## Conclusions

What are the conclusions regarding dose and timing in TMZ-radiotherapy? If we translate these considerations into the clinical application, we would like to suggest modification of the therapy protocol in a following way.

a) Given the facts that IR inhibits DNA synthesis and TMZ requires DNA replication, it is reasonable to conclude that the TMZ treatment should initially be carried out for 3 days without radiotherapy. This TMZ boost is anticipated to be most effective as it targets the proliferating glioblastoma cell population. For this boost a high dose (200 mg/m^2^/day), which is usually used for maintenance therapy, is recommended since it is expected to be effective also in cancer cells expressing MGMT at a very low level.
b) We further recommend starting the radio-chemotherapy cycle with a high dose of TMZ (200 mg/m^2^ + 2 Gy) for a short period (3 days) to enable DSBs to be formed according to the model in **Figure 2D**. After this TMZ-IR boost, therapy according to the classical scheme (TMZ 75 mg/m^2^ + RT 2 Gy) should be continued. With this boost TMZ-radiotherapy it is anticipated that in the initial phase of treatment the tumor cell cytotoxicity through TMZ can fully be expressed.
c) Given the fact that TMZ pretreatment is most effective in exerting radiosensitization, TMZ should be administered 2-4 hours before irradiation (the peak plasma level after oral TMZ is reached after about 2 hours and DNA alkylation is at its maximum after 3-4 hours).
d) Not only IR, but also TMZ causes cell cycle arrest, which limits apoptosis induction. If this immediate-early effect is transient (in contrast to senescence), it is anticipated that therapy-free intervals (of 2 days) may enhance the tumor-cytotoxic effect because of replication recovery. At the same time limiting side effects (notably hematotoxicity) might be reduced because of MGMT restoration in the stem cell compartment. Of note, CD35+ hematopoetic stem cells contain very low MGMT levels (41).
e) A benefit of concomitant TMZ-radiotherapy was recorded not only for promoter methylated, but also unmethylated (MGMT+) cases (42). This supports the view that an MGMT-independent minor pathway does exist, which is stimulated in the concurrent TMZ-radiotherapy setting (outlined in **Figure 2D**). Thus, on the basis of this model, a benefit of concurrent TMZ-radiotherapy is expected independent of the MGMT status.
f) Both IR and TMZ are strong inducers of cellular senescence, causing an irreversible proliferation arrest. This will necessarily negate the cytotoxicity of TMZ, although attenuating (transiently) tumor growth. Thus, the role of senescence-associated secretory phenotype, exhibited by glioblastoma cells upon TMZ treatment (17), for the progression of the disease remains to be established and the use of senolytic drugs in glioblastoma radio-chemo therapy a reasonable challenge.

## Data Availability Statement

The original contributions presented in the study are included in the article, further inquiries can be directed to the corresponding author.

## Author Contributions

BK: conceptualization, data curation, supervision, funding acquisition, and writing of the original draft. LB: experiments, quantification of apoptosis and senescence. HS: discussion, critical reading and improvements. All authors read and approved the submitted version of the manuscript.

## Conflict of Interest

The authors declare that the research was conducted in the absence of any commercial or financial relationships that could be construed as a potential conflict of interest.

## Publisher’s Note

All claims expressed in this article are solely those of the authors and do not necessarily represent those of their affiliated organizations, or those of the publisher, the editors and the reviewers. Any product that may be evaluated in this article, or claim that may be made by its manufacturer, is not guaranteed or endorsed by the publisher.
